# Chronic Cardiovascular Disorders Associated With COVID-19: A Literature Review

**DOI:** 10.7759/cureus.93271

**Published:** 2025-09-26

**Authors:** Adam Dudek, Marcin Bursy, Wojciech Szkudlarek, Jan Linkiewicz, Zbigniew Fabiszewski, Piotr Starosta

**Affiliations:** 1 General Practice, University Hospital in Poznań, Poznań, POL; 2 Medicine, Medical Center HCP, Poznań, POL; 3 Medical School, Poznań University of Medical Sciences, Poznań, POL; 4 General Surgery, Independent Healthcare Facility in Oborniki, Oborniki, POL; 5 General Practice, Independent Healthcare Facility in Oborniki, Oborniki, POL

**Keywords:** covid-19, covid-19 associated coagulopathy, covid-19 associated heart fibrosis, covid-19 associated hypertension, post covid syndrome

## Abstract

The severe acute respiratory syndrome coronavirus 2 (SARS-CoV-2), responsible for the COVID-19 pandemic, is now widely recognized for causing several long-term effects known as post-COVID-19 syndrome (PCS) or long COVID (LC). This presents a growing challenge for healthcare systems worldwide. This narrative review summarizes original peer-reviewed studies indexed in PubMed and published between January 2020 and August 2025. It focuses on adult populations unless stated otherwise. We included studies that provided primary clinical or imaging data on chronic cardiovascular outcomes after confirmed SARS-CoV-2 infection. We excluded case reports, pediatric-only cohorts, and non-peer-reviewed sources. Among the various cardiovascular issues related to LC, we focused on heart fibrosis (HF), postural orthostatic tachycardia syndrome (POTS), new-onset hypertension (HT), and coagulopathy. These conditions consistently show up in the reports and are significant in terms of illness, potential long-term disability, and public health impact. Although these issues are distinct in their underlying causes, they share common mechanisms. These include ongoing inflammation of the endothelium, disruption of the renin-angiotensin-aldosterone system (RAAS), immune-related tissue damage, and an ongoing state that promotes blood clots.

These processes can lead to measurable myocardial fibrosis that cardiac magnetic resonance imaging can detect, autonomic dysfunction often seen as POTS, a greater risk of developing hypertension shortly after infection, and a long-term rise in thromboembolic events due to increased clotting and resistant microclots. Current management is mostly focused on relief of symptoms and involves a team approach. It uses repurposed medications and tailored physical rehabilitation since no specific cure is available yet. Promising but still experimental methods, such as endothelial-protective agents like sulodexide and targeting inflammatory pathways, need thorough testing. There are significant gaps in our understanding of the long-term risk of hypertension, the natural progression of fibrosis, and the best treatment for POTS. This highlights urgent needs for future research. Beyond caring for individual patients, these ongoing cardiovascular problems raise important public health concerns. They include higher healthcare use, long-term disability, and economic costs. This situation requires increased clinical attention and proactive cardiovascular monitoring for those recovering from COVID-19.

## Introduction and background

Coronaviruses are a large and diverse family of RNA viruses. The virion particle consists of a capsid enveloped by a lipid envelope with multiple glycoproteins, which give coronaviruses a characteristic sun-like shape [1]. Coronaviruses usually cause intestinal and respiratory infections. Normally, the course of coronavirus infection is not severe, and is characterized by mild respiratory and intestinal symptoms like cough and diarrhoea. Sometimes, a viral infection can cause hepatitis and neurological illness [1]. However, there are some types of highly pathogenic coronaviruses, which were recognized as viruses causing severe acute respiratory disease (SARS) in 2002 and Middle East respiratory disease (MERS), which emerged in 2012. In December 2019, in Wuhan (China), a third highly pathogenic type of coronavirus, SARS coronavirus 2 (SARS-CoV-2), emerged [2].

The genome of SARS-CoV-2 is a single-stranded positive-sense RNA, and its genome is larger than that of any other RNA virus [1]. A new type of virus is highly transmissible by droplets, so it has quickly spread beyond China and caused a global pandemic. SARS-CoV-2 causes pneumonia; the course of infection is acute, accompanied by high fever, muscle pains, fatigue, and can lead to severe respiratory insufficiency. Patients with severe pulmonary syndromes often require artificial respiration support with the use of a respirator.

It has been observed that some patients who have recovered from the acute phase of COVID-19 develop a set of symptoms that persist over time despite the absence of detectable virus in the body [3].

This condition is defined by the World Health Organization (WHO) as “post-COVID condition” (PCC). According to WHO recommendations, PCC is diagnosed when symptoms occur three months after SARS-CoV-2 infection, persist for at least two months, and cannot be explained by other causes [4]. The US Department of Health and Human Services (HHS) calls this condition long COVID (LC), which is defined as symptoms that persist or appear after the initial COVID-19 infection and last longer than four weeks [5]. The UK National Institute for Health and Clinical Excellence (NICE) uses the name “post-COVID-19 syndrome” (PCS), which is diagnosed when symptoms occur after COVID-19 infection, persist for more than 12 weeks after the acute phase of infection, and cannot be explained by an alternative diagnosis [6]. Differences in diagnostic criteria between WHO, HHS, and NICE may influence the reported prevalence of LC and hinder direct comparison across studies. Variability in the minimum symptom duration and timing after infection introduces potential heterogeneity when interpreting the epidemiological data and clinical outcomes.

SARS-CoV-2 infection usually takes a milder form in younger patients without accompanying or chronic diseases; such patients recover faster. Moreover, in part of “low-risk patients," viral infection is asymptomatic [7]. However, patients with a mild course of the disease are more likely to develop PCS or LC.

A prospective observational study was conducted in 2020 on a group of 143 patients hospitalized due to confirmed SARS-CoV-2 infection [8]. The inclusion criterion for the study was virological recovery (negative real-time polymerase chain reaction (RT-PCR) test result). The average age of the subjects was 56 years, and the observation period lasted an average of 60.3 days (± 13.6 days) from the onset of the first symptoms of COVID-19. Well-being was assessed using a standardized questionnaire on symptoms and quality of life (assessed in comparison to the period before the onset of the disease). The study showed that two months after contracting COVID-19, as many as 87.4% of patients reported at least one persistent symptom, such as fatigue, shortness of breath, joint pain, etc. And 44.1% of those surveyed reported a deterioration in their quality of life compared to before they contracted COVID-19 [8]. The study showed that persistent symptoms after COVID-19 infection can be a serious problem for modern medicine. However, the study was limited by the relatively small and selective group of patients and the short observation period.

Although this review summarizes findings applicable to the general population, the primary focus is on adult patients, as the majority of available studies address adults, and pediatric data remain limited. Reports on children and adolescents are emerging but are still scarce and heterogeneous.

While coronaviruses have historically been associated with acute respiratory and intestinal infections, the SARS-CoV-2 pandemic revealed that a subset of patients experience persistent, multi-organ sequelae beyond the acute phase. Among these, cardiovascular complications have attracted particular attention due to their potential impact on morbidity and long-term health.

There are currently reports of chronic cardiovascular disorders that may be a consequence of COVID-19. Epidemiological estimates suggest that chronic cardiovascular manifestations occur in approximately 10-30% of individuals recovering from COVID-19, though reported prevalence varies depending on the definition of LC, population studied, and duration of follow-up. Suggested disorders include postural orthostatic tachycardia syndrome (POTS), coagulopathy, hypertension (HT), heart fibrosis (HF), etc. These symptoms often appear in populations that have had SARS-CoV-2 infection and had no previous risk factors for developing cardiovascular disorders. The direct mechanism of cardiological symptoms appears to be unknown yet. Proposed mechanisms include endothelial dysfunction, microvascular thrombosis, autoimmune activation, and direct cardiomyocyte injury mediated by the spike protein, as supported by early mechanistic and histopathological studies. Dysregulation of the autonomic nervous system has also been implicated in conditions such as POTS. However, some reasons may be connected with multi-organ damage called PCS, intensification of this damage, miscellaneous potential, and different recovery times of each tissue. Prolonged inflammation, immune response, cytokine storm, rare and hard-to-diagnose symptoms, a syndrome associated with intensive care or side effects of drugs, can be correlated with developing LC [9,10].

The aim of this paper is to review the literature on cardiovascular chronic complications caused by a past SARS-CoV-2 infection. The review was based on literature available on the PubMed platform, describing in particular POTS, HT, HF, coagulopathy associated with COVID-19 infection, and possible methods of their treatment. The review included original articles published between 2020 and 2025, available on the PubMed platform in August 2025. This narrative review was based on a targeted PubMed search and did not follow a formal systematic review protocol. Only peer-reviewed original articles in English were included, while preprints, case reports, and conference abstracts were excluded to ensure reliability of the evidence. The four cardiovascular complications, POTS, HT, HF, and coagulopathy, were selected because they represent the best-documented and clinically significant cardiovascular sequelae of LC in the current literature.

## Review

Heart fibrosis (HF)

COVID-19 has a strong correlation with long-term fibrosis of multisystem organs; also, the heart manifestation of HF could appear even after a long period of time after the initial infection [11,12]. The forms of manifestation are diverse and include symptoms such as cardiomegaly, tachycardia, or bradycardia [13].

There are many potential reasons for fibrosis, commonly associated with cytokine storm and an extended time of viremia connected to angiotensin-converting enzyme 2 (ACE-2) receptors, but also with hypertrophy of the left ventricular wall [14]. Another aspect is the role of pulmonary HT, and as a consequence of this clinical state, the development of right ventricular hypertrophy and fibrosis [15]. Impairment of the left ventricular function leads to noticeable symptoms, such as dyspnoea and breathlessness [16].

The most important tool in the diagnostic process of HF in LC is cardiac magnetic resonance (CMR). However, CMR has certain limitations, including difficulty distinguishing diffuse fibrosis from extracellular edema and reduced sensitivity for small subendocardial lesions. Standardized reference ranges and multimodal imaging approaches are a necessity in future diagnostics. It can be used both in cases of LC and in follow-up diagnostics of patients who have fully recovered from COVID-19 infection [17]. In a study, where the subjects were patients who had recently recovered from the SARS-CoV-2 infection in the year 2020, almost 80% of the whole group demonstrated some abnormal findings in the magnetic resonance imaging of the cardiac muscle. In the study by Puntmann et al., patients who had undergone CMR imaging shortly after recovering from a SARS-CoV-2 infection showed a common pattern of changes in the myocardium, such as raised native T1 and native T2. Both of these radiological findings suggest an inflammatory process within the structure of the myocardium. T1 is not a very specific parameter, as it can suggest both fibrotic changes and also edema, while a raised native T2 is less likely to be connected to fibrosis of the cardiac muscle. While raised native T1 and T2 values suggest involvement of the myocardium, T2 elevation typically reflects acute inflammatory edema, whereas late gadolinium enhancement is more indicative of fibrotic replacement tissue. Follow-up studies are necessary to confirm whether these changes persist and represent true fibrosis. Less than one-third of the patients were diagnosed with enhancement of the pericardium [17]. In patients with signs of PCS with symptoms of myocardial infection, findings suggesting fibrosis include a non-ischemic pattern of late gadolinium enhancement (LGE). Focal lesions with that characteristic have been found in 7% of all patients from the group presenting syndromes of PCS [18]. The symptoms are often nonspecific, for example, chest pain without typical characteristic radiation, as in coronary disease or pleural chest pain. Transmural fibrotic remodelling can be identified both in CMR and in echocardiography using the assessment of longitudinal strains in each region [12]. The specific region of the cardiac muscle or the quantified portion of the muscle afflicted with fibrotic remodelling has an impact on the contractility and basic function of the heart. This process, leading to ventricular hypertrophy, which is a relatively harmless consequence of postinfectious remodelling, can be completely asymptomatic and doesn't have an overly negative impact on the patient's well-being, or, as in this case, lead to exercise-induced chest pain [16]. Chronic heart failure as a consequence of fibrosis is progressing over time, so it requires multidisciplinary follow-up supervision. The most severe complications include ventricular tachycardia and acute heart failure [19]. Considering this data regarding the diagnostics in patients with PCS, cases with indications of irregular heart function and symptoms of heart failure should be evaluated using radiological tools, such as the CMR, as there is a proven way of utilizing the aforementioned parameters. In the study by Puntmann et al., CMR was performed at a median of 71 days after infection, allowing detection of subacute rather than purely acute changes. Longitudinal studies with imaging at 6-12 months are still limited but suggest persistence of fibrosis in a subset of patients [17]. In conclusion, it is important to highlight that patients with confirmed or suspected myocardial fibrosis may benefit from closer longitudinal monitoring, periodic echocardiographic strain analysis, and exercise prescriptions adjusted to the physical limitations each individual presents. Early initiation of guideline-directed medical therapy for heart failure caused by HF may be considered in those with reduced ejection fraction or high arrhythmic risk.

Postural orthostatic tachycardia syndrome (POTS)

In addition to the aforementioned symptoms in the course of LC, in some cases, POTS may also occur. Diagnostic criteria of POTS include an increase in heart rate of ≥ 30 bpm or ≥40 bpm in adolescent patients (aged 12-19 years) within 10 minutes after taking a standing position. To diagnose POTS, tachycardia should not be associated with changes in systemic blood pressure (BP), symptoms of orthostatic intolerance for at least three months, and should not be associated with any other diseases [20]. To be related to SARS-CoV-2 infection, POTS has to appear >12 weeks after viral infection during the duration of LCS [21]. A tilt table test (TUT) is preferably performed during the diagnosis, as in some cases it can resolve doubts about an initial clinical diagnosis. A patient who showed symptoms of orthostatic hypotension and increased adrenergic response has been diagnosed with POTS after performing the tilt test [22]. In the course of POTS, patients usually report cognitive impairment, headache, palpitations, and presyncope [23]. The mechanism of POTS is not fully explained. Presently, it is considered that the etiology of POTS can be related to viral infections or other conditions, which involve an excessive activation of the immune system, α-receptor hyperactivity, and the presence of autoantibodies against receptor molecules on human cells [24-26]. Moreover, it can also be linked with altered angiotensin II (ANGII), inducible nitric oxide synthase (iNOS), aldosterone, and renin activity [27]. It is also believed that POTS can arise from diminished heart size and mass. An LC can also be associated with dysregulation of the autonomic nervous system, causing a functional dysregulation in various compartments of the organism, including the cardiac conduction system [28]. Physical exercises and non-pharmacological treatment with a focus on adequate fluid supply should be considered as the most effective measure in POTS therapy. As for pharmacological agents, ivabradine has been proven useful in this condition. In a case study, ivabradine was prescribed at a dose of 5 mg twice a day. A visible clinical improvement has been reported within 24 hours after treatment initiation [29]. Other known agents that have been showing positive results in studies are selective beta-blockers, e.g., bisoprolol [30]. Moreover, after diagnosing the disease, it should be checked whether the patient is taking any medications that can interact with each other or the function of the cardiac conduction system and lead to the onset of the symptoms the patient is reporting [21].

However, as mentioned before, the exact mechanism of LC POTS still has to be explained, and accurate treatment regimens must be developed [26]. General recommendations for POTS not related to COVID-19 infection may be helpful. A rare invasive method used in Japanese LC patients with POTS is epipharyngeal abrasive therapy. Epipharyngeal abrasive therapy (EAT) is a treatment typically used in chronic epipharyngitis that involves applying zinc chloride to the epipharyngeal mucosa. During four months of weekly EAT sessions, a significant clinical improvement has been achieved without the use of any concomitant treatment methods [31].

Hypertension (HT)

HT is the most common cardiovascular disorder in the world. According to estimates, it may affect 30-40% of the global population, but according to a 2023 WHO report, only 54% of patients with elevated BP have been diagnosed, 42% are undergoing treatment, and only 21% have achieved the therapeutic goal of adequate HT control [32]. This condition involves an increase in systemic arterial BP. European guidelines authorize the diagnosis of this condition when BP readings taken in a doctor's office exceed 140/90 mmHg, or when average readings taken at home exceed 135/85 mmHg [33]. However, diagnostic thresholds vary internationally; for example, American guidelines define HT at ≥130/80 mmHg, and some of the cited studies used different settings of BP measurement (office, home, or ambulatory). This heterogeneity should be considered when comparing results across cohorts. Untreated or improperly treated HT is a risk factor for systemic complications such as heart failure, kidney failure, visual impairment, etc., and is also a factor contributing to increased mortality from cardiovascular disease [33].

The etiology of HT is complex and multifactorial, and recent scientific reports suggest that HT may be one of the manifestations of PCS. Importantly, most studies did not differentiate clearly between new-onset HT and worsening of pre-existing disease, which limits the interpretation of incidence and mechanisms.

During SARS-CoV-2 infection, the viral S-protein binds to ACE2, an enzyme commonly found on the cell membranes of the intestines, heart, kidneys, and blood vessel endothelial cells (EC). Physiologically, the product of the ACE2 enzymatic reaction, ANGII, is part of the renin-angiotensin-aldosterone system (RAAS), which plays a key role in maintaining adequate blood volume, electrolyte balance, and normal BP. ANGII acts on two types of receptors: type 1 (AT1R), whose stimulation leads to vasoconstriction, endothelial inflammatory response, and endothelial damage, and causes myocardial fibrosis and hypertrophy. Stimulation of the angiotensin II type 2 receptor (AT2R) promotes vasodilation, reduces platelet aggregation, and has a cardioprotective effect. Infection with the SARS-CoV-2 virus leads to a decrease in ACE2 expression, consequently leading to a shift in the balance in favor of a stronger effect on AT1R than AT2R. This leads to increased vasoconstriction, sodium and water retention, and intimal fibrosis, resulting in an increase in BP [34]. In addition, long-term inflammation of the endothelium caused by a previous SARS-CoV-2 infection may lead to the formation of pro-inflammatory factors such as tumor necrosis factor alpha (TNF-α) and interleukin 6 (IL-6), which accelerate endothelial fibrosis. Persistent inflammation also causes a decrease in nitric oxide production (which has a vasodilatory effect) [34]. Experimental data from animal and molecular studies further support these mechanisms, demonstrating SARS-CoV-2-induced ACE2 down-regulation, RAAS activation, endothelial fibrosis, and reduced nitric oxide bioavailability. All these factors may contribute to an increased risk of developing HT in patients with PCS.

In a retrospective cohort study from 2021, researchers compared the incidence of cardiac complications in patients after COVID-19. The study group consisted of 266,586 people aged 18 to 65 with a confirmed COVID-19 infection, confirmed by PCR or antigen testing. These individuals were observed for four months starting from the 21st day of infection. The results of the observation were compared to 8,980,919 medical records from 2020 (uninfected but with data in the system), 9,722,381 medical records from 2019 (historical, pre-pandemic cohort), and 1,655,907 medical records of people with lower respiratory tract infections (non-COVID LRTI). The study showed that the relative risk of developing HT in the course of PCS was 1.2-1.5, depending on the comparison group. It was particularly pronounced in the 18-34 age group [35]. Since follow-up lasted only four months, the persistence of this risk over longer periods remains uncertain.

The study from 2023 included 248 patients out of 393 patients diagnosed with COVID-19 and hospitalized at Civil Hospital Campus, Asarwa, between March 27, 2021, and May 27, 2021, in the study, who were followed up for one year for the development of LC complications. The results were compared to a healthy population. During the analysis, it was observed that 32.3% of the study participants developed HT [36]. This single-center study focused on hospitalized patients, who may carry a different baseline cardiovascular risk than non-hospitalized cohorts.

A 2023 meta-analysis analyzed five studies with a total of 19,293,346 clinical cases, of which 758,698 were people who had recovered from COVID-19 [37]. The average age of the patients studied was 54.6 years. In the publication analyzed, the average observation period was 6.8 months. The observed relative risk of HT was 1.7 (p < 0.0001) for the group diagnosed with SARS-CoV-2 infection. A higher risk of developing HT was also observed with increasing age of the subjects (p = 0.001). Female sex emerged as an independent risk factor (p = 0.03), although confirmation of this association in other populations is still limited. It was also observed that the risk of developing HT was highest in the first months after COVID-19, then decreased (p < 0.0001) [37]. The results indicate the need to monitor BP after COVID-19, especially in the first months after the infection. Nevertheless, studies should be conducted that involve long-term observation of these patients in order to collect reliable data on the long-term risk of developing HT as a part of PCS.

A retrospective cohort study from 2021, which analysed the electronic database of the US Veterans Affairs system, included two groups of patients [38]. The study group consisted of 73,435 patients with confirmed SARS-CoV-2, none of whom were hospitalized during the acute phase. The control group consisted of 4,990,835 patients without COVID-19. The average age was 60.7 years; 87.9% of the subjects were men. The median observation period was 126 days. Cox models were used to assess hazard ratios (HR), which ranged from 1.3 to 1.4 for the population with a history of SARS-CoV-2 infection. The inclusion of only non-hospitalized individuals highlights potential differences in risk depending on the severity of the acute infection [38]. The results indicate a correlation between COVID-19 infection and the development of HT. However, due to the limited duration of the study, long-term follow-up should be conducted.

HT caused by LC disorders is gaining increasing attention in the medical world. Attention is drawn to the relatively high incidence of this condition among patients who have had COVID-19. Because potential confounding factors such as corticosteroid use, obesity, or pre-existing cardiovascular risk were not uniformly controlled across studies, the true magnitude of risk remains uncertain. Nevertheless, there is still a lack of clear recommendations for screening for this condition in patients who have had SARS-CoV-2 infection and for therapeutic recommendations. Until disease-specific evidence emerges, management of PCS-related HT should follow current guideline-directed therapy for primary HT, and the development of standardized post-COVID BP monitoring protocols is strongly warranted. Therefore, further research is needed, including both meta-analyses of available publications and original studies, on the basis of which it would be possible to issue clear recommendations for the management of HT as a component of LC.

Coagulopathy

The acute phase SARS-CoV-2 infection in some cases predisposes to a procoagulation state. The coagulopathy associated with SARS-CoV-2 infection differs across disease phases. In the acute stage, systemic inflammation and sepsis-like coagulopathy predominate. In the subacute convalescent phase, markers of endothelial dysfunction and impaired fibrinolysis often persist. In the chronic stage, defined as LC, studies indicate the presence of fibrinolysis-resistant microclots, sustained endothelial activation, and a prolonged risk of thromboembolic complications. In this review, we therefore distinguish more clearly between data derived from acute COVID-19 cohorts, autopsy series, and long-term LC populations. This process can be characterized mainly by systemic inflammation and hypercoagulation dysregulation; The balance between the above-mentioned processes in the state of health is part of normal haemostasis. Dysregulation can lead to thrombotic incidents, especially in the long-term increase of proinflammatory cytokines. Infection with the SARS-CoV-2 virus causes an imbalance in the physiological balance of haemostasis and shifts it towards clot formation. Tissue factor (TF) plays a particularly important role in this context, as its expression is increased in activated monocytes and EC, which initiates excessive thrombin generation and promotes thrombus formation in micro- and macrocirculation. At the same time, there are disturbances in the anticoagulant systems - the activity of protein C and antithrombin is reduced, while the level of plasminogen activator inhibitor-1 (PAI-1) is increased, which leads to impaired fibrinolysis and thrombus stabilization [39]. In addition, interactions between inflammatory processes and coagulation intensify. These involve, among others, coagulation proteases such as thrombin and factor Xa. These compounds activate EC and express adhesive molecules, facilitating the adhesion and migration of leukocytes. During viral infection, the secretion of pro-inflammatory cytokines, especially IL-6 and TNF-α, increases. The cytokine storm caused by COVID-19 infection intensifies TF expression and platelet activation, thereby reinforcing the positive feedback loop between inflammation and coagulation [39]. Platelets deserve special attention as they play a particular role in this process. Under the influence of the above-mentioned inflammatory mediators, they release procoagulant substances such as thromboxane A2, which further intensifies thrombus formation. In addition, platelets activated in the coagulation process participate in leukocyte recruitment and regulation of endothelial permeability, promoting vascular damage and perpetuating the inflammatory response. As a result, a chronic thrombo-inflammatory spiral develops, which is the basis for thromboembolic complications and may play an important role in the pathogenesis of long-term LC symptoms [39].

The SARS-CoV-2 spike protein, and in particular its S1 subunit, plays a special role in the pathogenesis of these disorders. In addition to binding to the ATR-2 receptor and facilitating the entry of the virus into host cells, this protein can also interact directly with platelets and fibrinogen. This interaction leads to the activation of platelets, causing their uncontrolled activation and exacerbation of the prothrombotic state. Recent studies have shown that the S1 subunit has the ability to modify the structure of fibrin fibres, causing the formation of poorly soluble aggregates, which subsequently impede the process of fibrinolysis and the maintenance of haemostasis balance [39]. An increased proportion of substances such as von Willebrand factor (vWF), serum amyloid A (SAA), and various classes of antibodies has also been observed in microthrombi in patients with LC. Their presence further exacerbates persistent inflammation and haemostasis dysregulation. Due to their resistance to degradation, these microthrombi can remain in circulation for a long time, causing impaired tissue perfusion. As a result, chronic hypoxia occurs due to limited oxygen and nutrient supply, leading to organ dysfunction and symptoms characteristic of LC that persist for many months, such as chronic fatigue, cardiovascular disorders, decreased exercise tolerance, etc. [39].

In a publication by Pretorius et al. (2022), 80 people with persistent symptoms such as shortness of breath, fatigue, etc., after SARS-CoV-2 infection were analyzed [40]. Blood samples were taken from the study participants and analyzed using biochemical and microscopic methods to assess endothelial markers and the structure and function of platelets and fibrinogen. The results obtained were compared to those of individuals who had never tested positive for COVID-19. It was observed that patients complaining of symptomatic LC had statistically significantly elevated levels of markers of EC damage and activation, such as vWF and factor VIII. They also had significantly elevated levels of molecules responsible for regulating vascular wall tension and angiogenesis processes: endothelin-1 (ET-1) and agniopoetin-2 [40]. Furthermore, microthrombi with an amyloid structure, resistant to fibrinolysis, were observed in their plasma, which were not present in the control group [40]. The abnormalities detected in the study may be responsible for chronic microcirculation disorders and coagulopathy, which can lead to chronic symptoms in patients with LC. Nevertheless, although the study was conducted on a relatively large group of patients, it was quite uniform in terms of patient profiles. Furthermore, it was an observational cross-sectional study, which makes it difficult to establish a clear link between the detected biomarkers and clinical symptoms. Therefore, further studies on a larger and more diverse group of patients are required.

The other study involved histopathological and ultrastructural analysis of endothelial damage (endotheliitis) in patients who died from COVID-19 [41]. Autopsy and histopathological specimens from patients who died from SARS-CoV-2 infection were analyzed. The specimens were stained using classical histopathological techniques, and immunohistochemical techniques were also used to detect the SARS-CoV-2 virus in EC, as well as electron microscopy techniques. The samples came from organs such as the lungs, kidneys, and small intestine of patients from various European countries, such as Italy, Switzerland, etc. SARS-CoV-2 virus particles were detected in the tested preparations, and signs of inflammation, oedema, and EC apoptosis were observed, as well as the presence of microthrombi [41]. Nevertheless, the study was descriptive in nature and did not include a control group, so it is impossible to determine unequivocally whether these changes were a component of LC or the result of a viral infection. Further analysis of this topic is needed.

In a prospective observational study from 2021, patients with LC were evaluated in comparison with a healthy control group, examining microcirculation and endothelial biomarkers (vWF, factor VIII, ET-1, angiopoietin-2). The study group consisted of individuals complaining of LC symptoms after COVID-19, and the control group consisted of healthy individuals. The results showed chronic endothelial dysfunction and microcirculation disorders up to eight months after acute infection, suggesting a role for vascular damage in the persistence of PASC symptoms [42].

Studies demonstrated that in severe COVID-19, antibodies can induce the formation of so-called procoagulant platelets, which expose membrane phospholipids, creating a catalytic surface for coagulation factors and intensifying thrombin production [43]. The analyses were performed using flow cytometry and in vitro coagulation tests on plasma samples. The study group consisted of patients hospitalized with severe COVID-19, while the control group consisted of healthy donors and individuals hospitalized for other reasons, without SARS-CoV-2 infection. This phenomenon contributes to an increase in D-dimer concentration and promotes thrombosis, combining immunological mechanisms with the coagulopathy characteristic of COVID-19.

Moreover, phospholipid autoantibodies have been recognized as an important factor promoting thrombosis in SARS-CoV-2. They can reduce the anti-inflammatory and vasodilatory effects of nitrogen oxide (NO) by decreasing its concentration as a result of binding to EC receptors [44]. This study requires in-depth analysis.

Recent studies have shown a significant increase in thromboembolic events following COVID-19 infection. Risk factors for first arterial thrombosis and venous thrombosis among 48 million adults in England and Wales showed that in the week following COVID-19 infection, they were 28.7 and 33.2 times higher, respectively, than in people without a diagnosis of COVID-19. This risk decreased over time but remained elevated up to 49 weeks after diagnosis [45]. The study highlights the long-term prothrombotic effects of COVID-19, confirming the need to monitor patients even after the symptoms of acute infection have subsided. Population-based data confirm that thromboembolic risk remains clinically significant well beyond the acute infection. In the study of 48 million individuals in England and Wales, the risk of venous and arterial thrombosis remained elevated for up to 49 weeks post-infection [45]. These findings underscore that prothrombotic alterations in LC are not rare epiphenomena but represent a sustained and clinically important risk.

In another study, an analysis of 11.7 million veterans in the US, including more than 153,000 with SARS-CoV-2 infection, showed an increased risk of adverse cardiovascular outcomes, including a higher rate of myocardial infarction, acute coronary syndrome, pulmonary embolism, and stroke one year after infection [46]. The study demonstrated the necessity of further observation of patients after COVID-19 in order to quickly detect cardiac complications.

In a study conducted among 150 patients with COVID-19 at a median of 80 days after diagnosis, both hospitalized and outpatient, the mean D-dimer concentration was 327 ng/mL, with 25% of patients showing abnormally elevated values [47]. D-dimer levels three and six months after the acute phase were also checked by Kalaivani and Dinakar. After three months, 42% of patients had elevated (>500 ng/mL) D-dimer levels, with a decrease to 32% after six months [48]. Fan et al. (2022) reported a mean follow-up period of approximately 12 months among 39 patients, only nine of whom had severe disease. Compared to the control group, these patients had significantly higher D-dimer levels and factor VIII activity and significantly lower AT activity. Thrombin generation and markers of endotheliopathy were significantly higher in patients with LC [49]. All of those three studies indicate the potential usefulness of measuring D-dimer concentrations in patients with suspected LC for diagnostic purposes [47-49]. Nevertheless, verification of the D-dimer concentration ranges that enable LC detection is required through in-depth analysis. Although persistent D-dimer elevation has been observed in several LC cohorts, this biomarker suffers from limited specificity. D-dimer is elevated in numerous conditions, including malignancy, infection, and systemic inflammation, and threshold values vary across populations and assay platforms. No diagnostic cut-off has yet been validated to distinguish LC-related coagulopathy from other causes of elevated D-dimer.

After two months of observation in the study by Gerotziafas et al. and after four months in a series of 29 patients described by von Meijenfeldt et al., persistent hypercoagulability was found in individuals recovering from COVID-19. This phenomenon was characterized by increased thrombin generation and inhibition of fibrinolysis, which was associated with elevated levels of PAI-1 [50,51]. These results suggest that haemostatic imbalances may persist for many months after SARS-CoV-2 infection, even in patients who did not require hospitalisation or had a mild course of the disease. Mechanistically, chronic activation of EC and elevated PAI-1 concentrations may promote microthrombus formation and impede thrombus breakdown, which in turn increases the risk of late thromboembolic complications. These observations indicate the need to monitor coagulation parameters in individuals with LC, especially those with additional risk factors for thrombosis, and to consider possible anticoagulant prophylaxis in selected cases.

The role of anticoagulant or antiplatelet therapy in LC remains undefined. To date, no randomized controlled trials have formally assessed long-term prophylaxis in LC populations. Current recommendations emphasize individualized risk assessment, considering comorbidities, prior thrombotic events, and persistent symptoms, rather than universal prophylaxis.

A major limitation of the current evidence base is the heterogeneity of available studies. Cohorts differ in patient selection (hospitalized vs. ambulatory, severe vs. mild acute disease), follow-up duration (ranging from weeks to more than one year), and laboratory or imaging methodologies. This variability complicates direct comparisons and contributes to conflicting findings.

Future research should prioritize large prospective studies with standardized coagulation and endothelial panels to clarify the prevalence and mechanisms of persistent coagulopathy in LC. Novel imaging techniques, such as advanced microvascular imaging, may also provide non-invasive tools to assess microthrombi in vivo. Such approaches are essential to validate current mechanistic hypotheses and to translate them into targeted diagnostics and therapies.

Therapy

At this moment, there is no specific therapy to address the causes of LC. Treatment is mostly symptomatic and based on well-known FDA-approved drugs. One of the potential approaches for LC therapy is multidisciplinary care, which includes the provision of healthcare after patient discharge. New guidelines recommend tailored interventions depending on the patient’s condition, and a key issue is ensuring standardized therapy is equally applied.

There are promising data on the use of sulodexide as a drug supporting endothelial function. The study involved 410 patients. Patients were enrolled on average 1.89 ± 1.2 months after COVID-19 infection. At the time of enrollment, 210 (51.2%) patients had an Endothelial Quality Index (EQI) < 2. Of these, only 79 patients received sulodexide. Patients in the sulodexide group had lower baseline EQI than the non-medical intervention group (0.94 ± 0.6 vs. 1.52 ± 0.4; P < 10^-3^) and were more prone to diabetes, HT, and coronary artery disease, and took more long-term medications (aspirin, beta-blockers, and statins; P = 0.01, 0.002, 0.01, 0.009, 0.001, and 0.01, respectively) [52]. During the 21-day follow-up, patients receiving sulodexide reported fewer LC symptoms, particularly chest pain, palpitations, fatigue, and neurocognitive difficulties, along with a significant improvement in endothelial function (ΔEQI 1.26 ± 1.07 vs. 0.22 ± 0.7; P < 10^−3^) [52]. These results are preliminary, limited by the small treated subgroup and differences in comorbidities, highlighting the need for larger, controlled trials.

Another potential therapeutic target is the cannabinoid receptor type 1 (CB1) [53]. CB1 is activated by endocannabinoids, which are observed during COVID-19 infection. Its activation promotes a pro-inflammatory and pro-fibrotic state, contributing to fibroproliferation in the heart and potentially affecting lungs and kidneys. Inhibition of CB1 could prevent these changes. CB1 receptors are G-protein-coupled receptors abundant in neurons, modulating neurotransmission [53]. It should be noted that these findings are preclinical, and no human trials have confirmed their clinical applicability.

Similarly, iNOS is an enzyme involved in generating reactive nitrogen species, contributing to inflammation and acute respiratory distress syndrome (ARDS), which can predispose patients to long-term health complications [53]. Theoretical combined inhibition of iNOS and CB1 could reduce inflammation and fibrosis in the heart, lungs, and kidneys in PCC. This remains a hypothesis requiring validation in clinical trials with standardized, adequately sized patient cohorts [53].

Heparin-induced extracorporeal lipoprotein apheresis (HELP) effectively reduces hypercoagulability and inflammatory molecules in cardiovascular diseases [54]. While there is a pathophysiological rationale for its potential use in LC, it has not been tested in this population, is expensive, and has limited accessibility. Further studies are required to evaluate its safety and efficacy in LC [54].

Physical training and respiratory muscle exercises are highlighted as beneficial in LC. A meta-analysis by Tan et al. (2025) provides important information on the effectiveness of various treatments for LC [55]. The most promising forms of therapy are physical training, which improves exercise capacity and cardiopulmonary function, and respiratory muscle training. In cases of chronic fatigue, transcranial direct current stimulation (tDCS) may be helpful, although more research is needed. Palmitoylethanolamide-luteolin (PEA-LUT) supplementation may support recovery from olfactory disorders [55]. Patient selection, exercise intensity, and monitoring are important, particularly for those with post-exertional malaise.

In summary, no therapy currently addresses the causes of LC, and treatment remains largely symptomatic. Evidence supports the potential benefit of multidisciplinary strategies, sulodexide for endothelial function, and exercise-based interventions. CB1 and iNOS inhibition, as well as HELP therapy, remain experimental. Further prospective, randomized, placebo-controlled trials are necessary to evaluate the efficacy, safety, and impact on quality of life in LC patients [52-55].

## Conclusions

This comprehensive review underscores the profound and lasting impact of SARS-CoV-2 infection on the cardiovascular system, establishing LC as a significant risk factor for the new onset of chronic cardiovascular diseases. The evidence presented reveals that the pathophysiological mechanisms, primarily persistent endothelial dysfunction, chronic inflammation, immune dysregulation, and a pro-thrombotic state, converge to drive a spectrum of complications, including myocardial fibrosis, POTS, new-onset HT, and a sustained hypercoagulable condition. Some of these findings are derived from observational studies, and heterogeneity of study populations, as well as differences in follow-up duration, need to be acknowledged when interpreting the results. A critical finding is that these complications are not exclusive to patients who suffered severe acute COVID-19, but also mild infections. Large-scale epidemiological studies consistently show a significantly elevated risk for these conditions, particularly in the first months following infection, highlighting the necessity for heightened clinical vigilance. It remains uncertain whether this risk plateaus over time or persists long-term, and more standardized and prolonged observation will be required to clarify this trajectory. From a clinical perspective, these insights call for structured cardiovascular risk stratification and the use of selected biomarkers and imaging tools to guide monitoring. Periodic screening, especially within the first year after infection, may be warranted, with particular attention to younger patients and women. Healthcare systems should adapt by integrating LC cardiovascular care into existing infrastructures, combining cardiology, rehabilitation, and primary care services. Consideration must also be given to cost-effectiveness and resource implications, as widespread long-term monitoring will inevitably challenge health systems already under strain. At present, the therapeutic landscape for PCS-related cardiovascular sequelae remains challenging. Management is predominantly symptomatic and based on multidisciplinary care. Exercise-based rehabilitation requires careful patient selection, with intensity adjusted individually and safety closely monitored, particularly in patients with post-exertional malaise, where overexertion can exacerbate symptoms. While promising experimental approaches are emerging, including CB1 receptor antagonism, iNOS inhibition, and lipid apheresis, their use in LC is still theoretical. To date, there are no clinical trial data in humans with LC, and most evidence comes from preclinical studies or extrapolation from other cardiovascular conditions. Potential adverse effects must be carefully evaluated.

In summary, SARS-CoV-2 has unveiled a new frontier in cardiovascular medicine. The focus must shift from managing the acute infection to addressing its long-term consequences. Proactive screening, comprehensive patient care, and dedicated research are essential. Research priorities should include randomized controlled trials, standardized diagnostic criteria, and long-term registries to provide reliable evidence. Only through such measures will it be possible to mitigate the growing burden of LC-related cardiovascular disease on individuals and on healthcare systems worldwide.

## References

[1] V'kovski P, Kratzel A, Steiner S, Stalder H, Thiel V (2021). Coronavirus biology and replication: implications for SARS-CoV-2. Nat Rev Microbiol.

[2] Jackson CB, Farzan M, Chen B, Choe H (2022). Mechanisms of SARS-CoV-2 entry into cells. Nat Rev Mol Cell Biol.

[3] Scholkmann F, May CA (2023). COVID-19, post-acute COVID-19 syndrome (PACS, "long COVID") and post-COVID-19 vaccination syndrome (PCVS, "post-COVIDvac-syndrome"): similarities and differences. Pathol Res Pract.

[4] (2025). COVID-19 cases, world. https://covid19.who.int/.

[5] Greenhalgh T, Sivan M, Perlowski A, Nikolich JŽ (2024). Long COVID: a clinical update. Lancet.

[6] (2020). COVID-19 rapid guideline: managing the long-term effects of COVID-19. https://www.nice.org.uk/guidance/ng188.

[7] Zsichla L, Müller V (2023). Risk factors of severe COVID-19: a review of host, viral and environmental factors. Viruses.

[8] Carfì A, Bernabei R, Landi F (2020). Persistent symptoms in patients after acute COVID-19. JAMA.

[9] Raman B, Bluemke DA, Lüscher TF, Neubauer S (2022). Long COVID: post-acute sequelae of COVID-19 with a cardiovascular focus. Eur Heart J.

[10] Gyöngyösi M, Alcaide P, Asselbergs FW (2023). Long COVID and the cardiovascular system-elucidating causes and cellular mechanisms in order to develop targeted diagnostic and therapeutic strategies: a joint scientific statement of the ESC Working Groups on cellular biology of the heart and myocardial and pericardial diseases. Cardiovasc Res.

[11] Nie X, Qian L, Sun R (2021). Multi-organ proteomic landscape of COVID-19 autopsies. Cell.

[12] Savchenko O, Tyravska Y, Moshkovska Y, Pliskevych D, Altunina N, Lizogub V (2023). A case of long COVID-19 myocarditis with asymptomatic manifestation in a young male. Galician Med J.

[13] Lampropoulos CE, Mavrogeni S, Dervas A (2021). Myocardial fibrosis after COVID-19 infection and severe sinus arrest episodes in an asymptomatic patient with mild sleep apnea syndrome: a case report and review of the literature. Respir Med Case Rep.

[14] Figueiro-Filho EA, Hobson SR, Farine D, Yudin MH (2021). Highly expressed ACE-2 receptors during pregnancy: a protective factor for SARS-COV-2 infection?. Med Hypotheses.

[15] Martínez-Mateo V, Fernández-Anguita MJ, Paule A (2020). Electrocardiographic signs of acute right ventricular hypertrophy in patients with COVID-19 pneumonia: a clinical case series. J Electrocardiol.

[16] Janonytė U, Cieškaitė SG, Sėlenytė M, Žebrauskaitė-Keblikienė G, Lapinskas T, Žaliaduonytė D (2025). Myocardial fibrosis in a recovered COVID-19 patient: a case indicating prior myocarditis. Cureus.

[17] Puntmann VO, Carerj ML, Wieters I (2020). Outcomes of cardiovascular magnetic resonance imaging in patients recently recovered from coronavirus disease 2019 (COVID-19). JAMA Cardiol.

[18] Kravchenko D, Isaak A, Zimmer S (2021). Cardiac MRI in patients with prolonged cardiorespiratory symptoms after mild to moderate COVID-19. Radiology.

[19] Elia S, De Vecchi S, D'Amario D, Patti G (2025). A case report of long COVID-19 and chronic active myocarditis. Eur Heart J Case Rep.

[20] Sheldon RS, Grubb BP 2nd, Olshansky B (2015). 2015 Heart Rhythm Society Expert Consensus Statement on the diagnosis and treatment of postural tachycardia syndrome, inappropriate sinus tachycardia, and vasovagal syncope. Heart Rhythm.

[21] Amekran Y, Damoun N, El Hangouche AJ (2022). Postural orthostatic tachycardia syndrome and post-acute COVID-19. Glob Cardiol Sci Pract.

[22] Johansson M, Ståhlberg M, Runold M (2021). Long-haul post-COVID-19 symptoms presenting as a variant of postural orthostatic tachycardia syndrome: the Swedish experience. JACC Case Rep.

[23] Diekman S, Chung T (2023). Post-acute sequelae of SARS-CoV-2 syndrome presenting as postural orthostatic tachycardia syndrome. Clin Exp Emerg Med.

[24] Wallukat G, Hohberger B, Wenzel K (2021). Functional autoantibodies against G-protein coupled receptors in patients with persistent Long-COVID-19 symptoms. J Transl Autoimmun.

[25] Miglis MG, Prieto T, Shaik R, Muppidi S, Sinn DI, Jaradeh S (2020). A case report of postural tachycardia syndrome after COVID-19. Clin Auton Res.

[26] Gall NP, James S, Kavi L (2022). Observational case series of postural tachycardia syndrome (PoTS) in post-COVID-19 patients. Br J Cardiol.

[27] Vernino S, Bourne KM, Stiles LE (2021). Postural orthostatic tachycardia syndrome (POTS): state of the science and clinical care from a 2019 National Institutes of Health Expert Consensus Meeting - part 1. Auton Neurosci.

[28] Sonel Tur B, Köseoğlu BF, Kutay Ordu Gökkaya N (2022). COVID-19, cardiac involvement and cardiac rehabilitation: insights from a rehabilitation perspective - state of the art. Turk J Phys Med Rehabil.

[29] O'Sullivan JS, Lyne A, Vaughan CJ (2021). COVID-19-induced postural orthostatic tachycardia syndrome treated with ivabradine. BMJ Case Rep.

[30] Tsuchida T, Ishibashi Y, Inoue Y (2024). Treatment of long COVID complicated by postural orthostatic tachycardia syndrome-case series research. J Gen Fam Med.

[31] Takezawa H (2025). Successful treatment of long-COVID postural tachycardia syndrome with epipharyngeal abrasive therapy in an adolescent patient: a case report. Medicine (Baltimore).

[32] Goorani S, Zangene S, Imig JD (2024). Hypertension: a continuing public healthcare issue. Int J Mol Sci.

[33] McEvoy JW, McCarthy CP, Bruno RM (2024). 2024 ESC guidelines for the management of elevated blood pressure and hypertension. Eur Heart J.

[34] Składanek JA, Leśkiewicz M, Gumiężna K (2024). Long COVID and its cardiovascular consequences: what is known?. Adv Clin Exp Med.

[35] Daugherty SE, Guo Y, Heath K (2021). Risk of clinical sequelae after the acute phase of SARS-CoV-2 infection: retrospective cohort study. BMJ.

[36] Vyas P, Joshi D, Sharma V, Parmar M, Vadodariya J, Patel K, Modi G (2023). Incidence and predictors of development of new onset hypertension post COVID-19 disease. Indian Heart J.

[37] Zuin M, Rigatelli G, Bilato C, Pasquetto G, Mazza A (2023). Risk of incident new-onset arterial hypertension after COVID-19 recovery: a systematic review and meta-analysis. High Blood Press Cardiovasc Prev.

[38] Al-Aly Z, Xie Y, Bowe B (2021). High-dimensional characterization of post-acute sequelae of COVID-19. Nature.

[39] Turner S, Khan MA, Putrino D, Woodcock A, Kell DB, Pretorius E (2023). Long COVID: pathophysiological factors and abnormalities of coagulation. Trends Endocrinol Metab.

[40] Pretorius E, Venter C, Laubscher GJ (2022). Prevalence of symptoms, comorbidities, fibrin amyloid microclots and platelet pathology in individuals with long COVID/post-acute sequelae of COVID-19 (PASC). Cardiovasc Diabetol.

[41] Varga Z, Flammer AJ, Steiger P (2020). Endothelial cell infection and endotheliitis in COVID-19. Lancet.

[42] Charfeddine S, Ibn Hadj Amor H, Jdidi J (2021). Long COVID 19 syndrome: is it related to microcirculation and endothelial dysfunction? Insights from TUN-EndCOV study. Front Cardiovasc Med.

[43] Althaus K, Marini I, Zlamal J (2021). Antibody-induced procoagulant platelets in severe COVID-19 infection. Blood.

[44] Knight JS, Caricchio R, Casanova JL (2021). The intersection of COVID-19 and autoimmunity. J Clin Invest.

[45] Knight R, Walker V, Ip S (2022). Association of COVID-19 with major arterial and venous thrombotic diseases: a population-wide cohort study of 48 million adults in England and Wales. Circulation.

[46] Xie Y, Xu E, Bowe B, Al-Aly Z (2022). Long-term cardiovascular outcomes of COVID-19. Nat Med.

[47] Townsend L, Fogarty H, Dyer A (2021). Prolonged elevation of D-dimer levels in convalescent COVID-19 patients is independent of the acute phase response. J Thromb Haemost.

[48] Kalaivani MK, Dinakar S (2022). Association between D-dimer levels and post-acute sequelae of SARS-CoV-2 in patients from a tertiary care center. Biomark Med.

[49] Fan BE, Wong SW, Sum CL (2022). Hypercoagulability, endotheliopathy, and inflammation approximating 1 year after recovery: assessing the long-term outcomes in COVID-19 patients. Am J Hematol.

[50] Gerotziafas GT, Van Dreden P, Sergentanis TN (2022). Persisting endothelial cell activation and hypercoagulability after COVID-19 recovery - the prospective observational ROADMAP-post COVID-19 study. Hemato.

[51] von Meijenfeldt FA, Thålin C, Lisman T (2021). Sustained prothrombotic changes in convalescent patients with COVID-19. Lancet Haematol.

[52] Charfeddine S, Ibn Hadjamor H, Torjmen S (2021). Sulodexide in the treatment of patients with long COVID 19 symptoms and endothelial dysfunction: the results of TUN-EndCOV study. Archives of Cardiovascular Diseases Supplements.

[53] Udi S, Hinden L, Ahmad M (2020). Dual inhibition of cannabinoid CB(1) receptor and inducible NOS attenuates obesity-induced chronic kidney disease. Br J Pharmacol.

[54] Jaeger BR, Arron HE, Kalka-Moll WM, Seidel D (2022). The potential of heparin-induced extracorporeal LDL/fibrinogen precipitation (H.E.L.P.)-apheresis for patients with severe acute or chronic COVID-19. Front Cardiovasc Med.

[55] Tan C, Meng J, Dai X (2025). Effects of therapeutic interventions on long COVID: a meta-analysis of randomized controlled trials. EClinicalMedicine.

